# *QuickStats:* Age-Adjusted* Alzheimer’s Disease Death Rates^†^ Among Persons Aged ≥65 Years, by State^§^ — United States, 2015

**DOI:** 10.15585/mmwr.mm6627a6

**Published:** 2017-07-14

**Figure:**
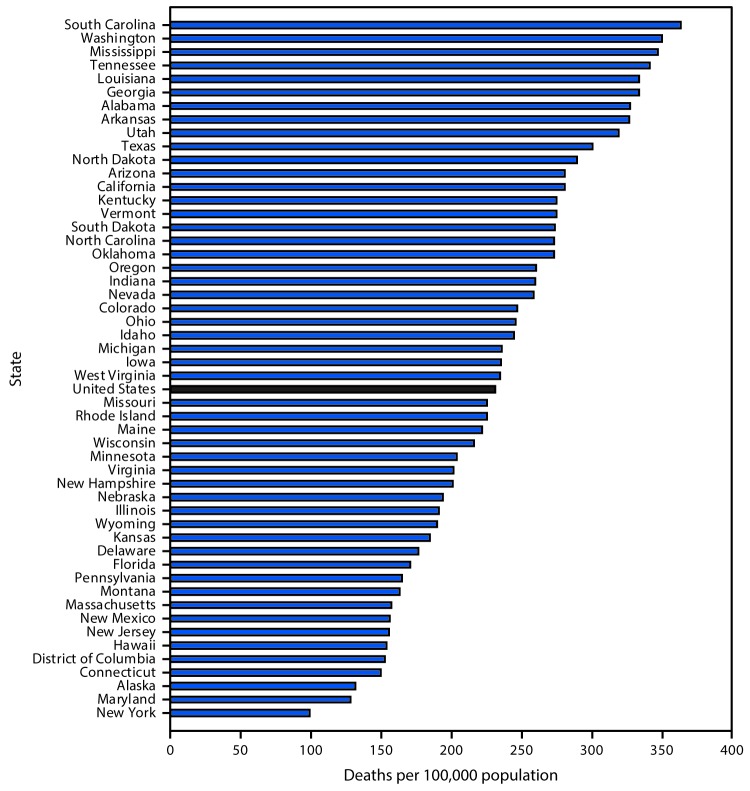
In 2015, the age-adjusted Alzheimer’s disease death rate among persons aged ≥65 years in the United States was 231.0 per 100,000 population. The five states with the highest age-adjusted death rates for Alzheimer’s disease were South Carolina (362.8), Washington (349.6), Mississippi (346.5), Tennessee (340.8), and Louisiana (333.6). New York had the lowest rate (99.0), followed by Maryland (128.2), Alaska (131.7), Connecticut (149.3), and the District of Columbia (152.2).

**Source:** National Vital Statistics System. Mortality public use data files, 2015. https://www.cdc.gov/nchs/data_access/vitalstatsonline.htm.

